# Influence of Inter- and Intra-Batch Variability on the Sample Size Required for Demonstration of Equivalent Microstructure of Semisolid Dosage Forms

**DOI:** 10.3390/pharmaceutics12121159

**Published:** 2020-11-28

**Authors:** Zhengguo Xu, Víctor Mangas-Sanjuán, Matilde Merino-Sanjuán, Virginia Merino, Alfredo García-Arieta

**Affiliations:** 1 Departamento de Farmacia y Tecnología Farmacéutica y Parasitología, Facultad de Farmacia, Universitat de València, Av. Vicente Andrés Estellés s/n, Burjassot, 46100 Valencia, Spain; zhengguoxu@gmail.com (Z.X.); victor.mangas@uv.es (V.M.-S.); Virginia.Merino@uv.es (V.M.); 2 Instituto Interuniversitario de Investigación de Reconocimiento Molecular y Desarrollo Tecnológico (IDM), Universitat Politècnica de València, Universitat de València, 46100 Valencia, Spain; 3 División de Farmacología y Evaluación Clínica, Departamento de Medicamentos de Uso Humano, Agencia Española de Medicamentos y Productos Sanitarios, Calle Campezo 1, Edificio 8, 28022 Madrid, Spain; agarciaa@aemps.es

**Keywords:** inter-batch variability, intra-batch variability, equivalence, topical products

## Abstract

Inter- and intra-batch variability of the quality attributes contribute to the uncertainty for demonstrating equivalent microstructure of post-approval changes and generic/hybrids of semisolid topical products. Selecting a representative sample size to describe accurately the in vitro properties of semisolids and to reach enough statistical power to demonstrate similarity between two semisolid topical products is currently challenging. The objective of this work is to establish the number of batches and units per batch to be compared based on different inter-batch and intra-batch variability to demonstrate equivalence in the physical characteristics of the products that ensure a similar microstructure of the semisolid. This investigation shows that the minimum number of batches to be compared of each product is 3 and the minimum number of units per batch could be 6 in the case of low intra- and inter-batch variability. If the products are not identical, i.e., 2.5–5% differences that are expected due to differences in the manufacturing process or the suppliers of excipients, 12 units and 6 batches are needed. If intra- or inter-batch variability is larger than 10%, the number of batches and/or the number of units needs to be increased. As the interplay between inter- and intra-batch variability is complex, the sample size required for each combination of inter- and intra-batch variability and expected difference between products can be obtained in the attached tables.

## 1. Introduction

The draft guideline on the quality and equivalence of topical products released by the European Medicines Agency (EMA) [1] states that the demonstration of therapeutic equivalence of two products requires comparison of their qualitative and quantitative composition, as well as a comparison of their physical properties, e.g., in case of post-approval changes or generic/hybrid products. According to this document: (1) the comparison of the physical properties should be conducted in at least three batches of the reference product and three batches of the test product with at least 12 replicates per batch, and (2) to consider equivalence for quantitative quality characteristics, the 90% confidence interval (CI) for the difference of means of the test and comparator products should be contained within the acceptance criteria of ±10% of the comparator product mean, assuming normal distribution of data.

The demonstration of equivalence by in vitro testing, alone or in combinations with other studies, with at least 3 batches of test and 3 batches of reference is recommended also by the U.S. FDA in some product specific guidance for topical products (e.g., acyclovir ointment, fluorescein sodium and benoxinate hydrochloride solution, benzyl alcohol lotion, bexarotene gel, clindamycine phosphate foam and gels, clindamycin phosphate and tretinoin gels, crisaborole ointment, dapsone gels, docosanol cream, doxepin HCl cream, efinaconazole solution, glycopyrronium tosylate cloth, hydrogen peroxide solution, ivermectin cream and lotion, loteprednol etabonate gel and ointment, luliconazole cream, metronidazole creams and gel, oxymetazoline HCl cream, ozenoxacin cream, penciclovir cream, pimecrolimus cream, silver sulfadiazine cream, tacrolimus ointments, tobramycin ointment), where a population bioequivalence approach is recommended for the comparison of relevant physical and chemical properties such as the particle size distribution of suspensions [2,3,4,5,6]. In the case of other dosage forms, no fewer than 10 units from each batch are required (e.g., injectable emulsions, nasal and inhalation products), but a minimum number of replicates is not defined for these topical products [7,8].

Sample size calculation is necessary for a correct demonstration of in vitro equivalence when a waiver of in vivo studies is applied. This comparability exercise has to be based on a protocol, where the objective of the comparison is defined (i.e., to obtain a biowaiver), as well as the in vitro tests to be conducted to conclude in vitro similarity, the statistical methods to be employed in the comparisons, and the acceptance range, which is 10% by default in some of the EMA guidelines, unless otherwise justified [9,10]. Unfortunately, it is observed too frequently that these comparisons are conducted in an exploratory manner as part of the pharmaceutical development. Those preliminary investigations could be considered as pilot studies from which the sponsor can obtain the expected difference between test and reference and the estimations of the expected intra-batch and inter-batch variability of test and reference product. Importantly, small pilot studies are known to underestimate the variance in clinical trials [11] and the upper boundary of the confidence interval of the variance estimation has been proposed for the sample size calculation [12]. Thereafter, a confirmatory study should be conducted with a protocol and a proper sample size calculation.

Rheological parameters, among others, are representative of the microstructure of topical products [13]. The EMA draft guideline specifies that when a product has non-Newtonian rheological behavior the complete flow curve (viscosity or shear stress versus shear rate) and the linear viscoelastic response (creep-recovery tests or oscillatory measurements at different frequencies) have to be characterized. From these, assessment parameters as viscosities at specified shear rates, yield stress values, thixotropic relative area, elastic and viscous moduli, or loss tangent can be calculated.

In a previous work [14,15] all these parameters were tested in ten batches of a reference topical product (Daivobet ointment containing 50 μg/g of calcipotriol and 0.5 mg/g of betamethasone dipropionate), in order to evaluate the feasibility to show equivalence between batches of the reference product within the limits proposed in the draft guideline with 12 replicates per batch and it was observed that the limits established in the guideline are too restrictive in case of rheological parameters with variability higher than 9.5%, even when 5 batches of each product under comparison, instead of 3, are tested. Equivalent microstructure between batches cannot be demonstrated with a reasonable sample size when the acceptance range was set at ±10%, since several rheological parameters exhibit inter-batch variability >10%. Therefore, either a wider fixed acceptance range or an acceptance range widened based on the inter-batch variability of the reference product were proposed as alternatives.

The objectives of this work are (i) to investigate the scenarios where the minimum sample size defined in the draft guideline is adequate and whether a smaller sample size might be acceptable in some cases, and (ii) to establish the number of batches and replicates per batch to be compared based on several scenarios of differences between test and reference products, inter-batch and intra-batch variability.

## 2. Materials and Methods

### 2.1. Study Design

The simulated scenarios were generated from the combination of the different variables specified below:

T/R: Test/Reference ratio (%): 100 (no difference), 97.5 (2.5% difference) and 95 (5% difference)
N_B_: Number of batches per product: 1, 2, 3, 6 and 12
N_U_: Number of units/replicates per batch: 6, 12 and 24
IBV: Inter-batch variability (%): 0, 2.5, 5, 7.5, 10, 15 and 20
ABV: Intra-batch variability (%): 0, 2.5, 5, 7.5, 10, 15 and 20

A total of 2205 scenarios were simulated, assuming 3 levels of T/R, 5 levels of N_B_ (1, 2, 3, 6 and 12), 3 levels of N_U_ (6, 12 and 24), 7 levels of IBV (0, 2.5, 5, 7.5, 10, 15 and 20), and 7 levels of ABV (0, 2.5, 5, 7.5, 10, 15 and 20). All study design conditions were arbitrary selected, but they were aimed to describe frequently observed experimental conditions. In addition, some of these scenarios provided the same total number of observations (e.g., 3 batches and 24 replicates per batch, 6 batches and 12 replicates per batch and 12 batches and 6 replicates per batch = 72 observations) to assess the better way to address the inter- and the intra-batch variability. The inter-batch and intra-batch variability encompass both low and high variability conditions observed in the literature [14,16,17,18,19] in order to evaluate the impact of variability on the probability to show equivalence depending on the simulated scenarios of difference between products and the number of batches and replicates.

### 2.2. Data Simulation

The simulation of a single experiment started with obtaining the population parameters of each product. The population value of the reference product (TVP_R_) was fixed to a value of 1 and the population value for the test product (TVP_T_) was established based on the T/R ratio considered in the simulation settings.

Then, parameter values for each batch of each product were randomly selected based on an exponential error model as follows:

$$P_{j_{k}}=TVP_{j}\cdot e^{\eta_{IBV}}$$

where *j* represents the reference or test product and *k* represents batch, $P_{j_{k}}$ is the parameter value for *k*th batch of reference or test product; and $\eta_{IBV}$ is the inter-batch variability, with mean zero value and variance $\omega_{IBV}^{2}$ ($\eta_{IBV}∼N\left(0,\omega_{IBV}^{2}\right)$). The inter-batch variance was derived from the coefficient of variation established for the IBV using the following equation:

$$\omega_{IBV}^{2}={\left(\frac{CV_{IBV}}{100}\right)}^{2}$$

Individual rheological parameters ($P_{j_{k_{i}}}$) for each batch were simulated also assuming an exponential model as follows:

$$P_{i_{j_{k}}}=P_{j_{k}}\cdot e^{\eta_{ABV}}$$

where *i* represents individual unit within batch *k* for product *j*; $\eta_{ABV}$ is the intra-batch variability, with mean zero value and variance $\omega_{ABV}^{2}$ ($\eta_{ABV}=N\left(0,\omega_{ABV}^{2}\right)$. The intra-batch variance was derived from the coefficient of variation established for the ABV using the following equation:

$$\omega_{ABV}^{2}={\left(\frac{CV_{ABV}}{100}\right)}^{2}$$

This process was repeated until 1000 experiments were simulated for each scenario in order to calculate the probability of similarity per scenario.

### 2.3. Bootstrap Analysis

A non-parametric bootstrap (*n* = 10,000) with replacement was conducted on each experiment per scenario based on the simulated data in order establish the bootstrap distribution of the parameter for each experiment of each scenario. Ninety percent non-parametric confidence intervals were calculated from the bootstrap distribution of the geometric mean ratios using the percentile method. Given the bootstrap distribution of the parameters, θ(*1), θ(*2),…,θ(*B), the percentile confidence interval is the interval between the 100·α and 100·(1 − α) quantiles of the bootstrap distribution, where α is the nominal type I error. Similarity was concluded when the non-parametric 90% confidence interval for the ratio test/reference was within the ±10% acceptance range (90.00–111.11%).

### 2.4. Software

R (V. 4.0.3, http://cran.r-project.org, GNU project) and R-studio (V.1.3.1093, Boston, MA, USA, 2019) were used for the bootstrap analyses and graphical display, respectively. All the analyses were performed on a Linux server from the University of Valencia (LluisVives) with Xeon 7500 series hexacore at 2.67 GHz and 2048 GB RAM.

## 3. Results

**Figure 1:**
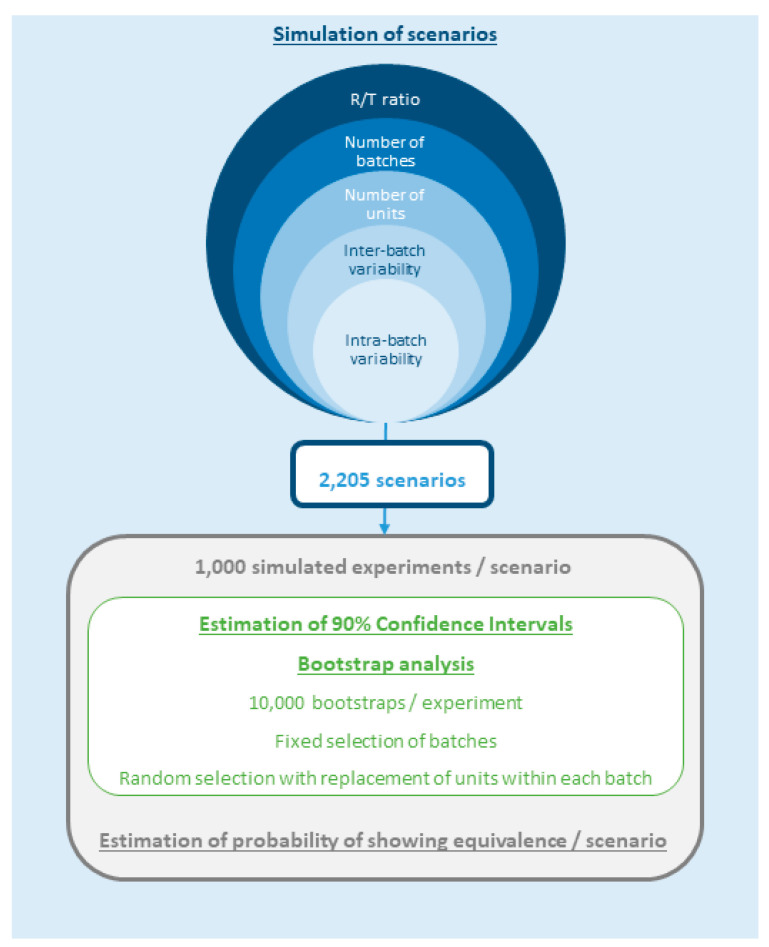
The simulation workflow applied to (i) simulate the scenarios, and (ii) estimate the probability of declaring equivalence per scenario.

Figure 1 depicts the simulation workflow followed to generate firstly the 2205 scenarios through the combination of different factors (T/R, N_B_, N_U_, IBV and ABV), and secondly, the estimation of the probability of showing equivalence through the stochastic simulation of 1000 experiments per scenario, including 10,000 bootstraps on each simulation in order to calculate the 90% CI of the T/R ratio.

Table 1, Table 2 and Table 3 summarize the probability of concluding similarity for each scenario, assuming different T/R ratios, N_B_, N_U_, IBV and ABV. These tables include the possibility of zero inter- and intra-batch variability, although not realistic, to show that bioequivalence is always shown in the absence of variability since the simulated differences are lower than 10%. These scenarios might represent scenarios with extremely low variability (<0.5%). In such cases, the sample size (i.e., N_B_ and N_U_) could be extremely low.

These simulations show that with the conditions required in the draft guideline (3 batches and 12 replicates), there is statistical power to conclude similarity only if the IBV and ABV does not exceed 7.5% if the products are equal, and 5% if the products are 2.5 or 5% different, which a usual assumption when calculating the sample size. If the IBV is low (e.g., 2.5%) there is at least 80% power in scenarios of more ABV (e.g., 15% if products are identical, 10% if the products differ only 2.5%, and 7.5% if the products differ 5%). Similarly, if the ABV is only 5%, there is at least 80% power to show similarity for IBV of 7.5% when the products differ 2.5%, but 5% IBV if the products differ 5% in their mean values. Therefore, the number of batches or the number of replicates have to be increased when the variabilities are higher than 7.5%, or even 5% if the products are not identical.

Table 1, Table 2 and Table 3 also show the probability of success when only one or two batches are tested. Importantly, the investigation of only one batch of test and one batch of reference and 6 units per batch is reasonable only where the products are identical, the ABV is ≤5% and the IBV is ≤2.5%. If the products differ 2.5%, the ABV and the IBV cannot exceed 2.5%. If 12 units are tested per batch, 80% power is achieved in the case of 7.5% intra- and 2.5% inter-batch variability, which illustrates that the ABV has to be compensated by increasing the number of units per batch, and 81% power is achieved in case of 5% of IBV and 2.5% ABV, which illustrates that the increase in the total sample size is also useful to compensate the increase in IBV.

**Figure 2:**
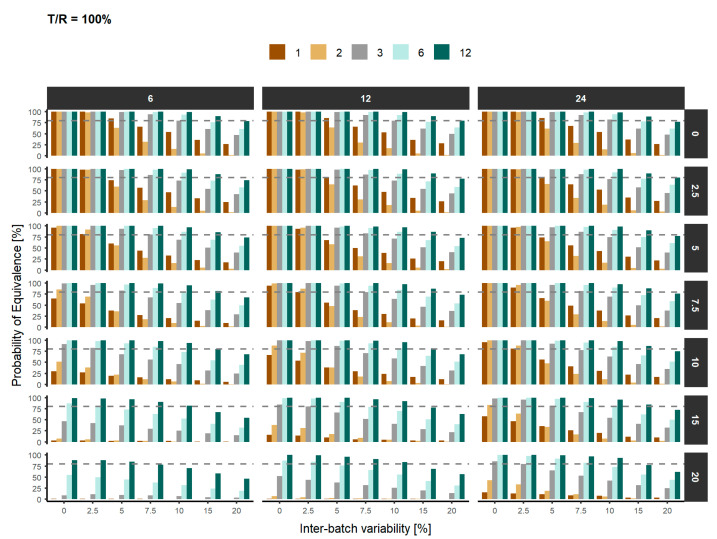
Probability of declaring similarity based on the inter-batch variability and the number of batches (brown: 1, gold: 2, grey: 3, light green: 6 and dark green: 12). Top panels represent the number of units and right panels represent the intra-batch variability. The grey dashed line represents the statistical power to declare equivalence between both products (80%).

Figure 2 represents the probability of declaring similarity when the T/R ratio is equal to 100% based on the IBV. The results are stratified by the number of units and the ABV considered.

**Figure 3:**
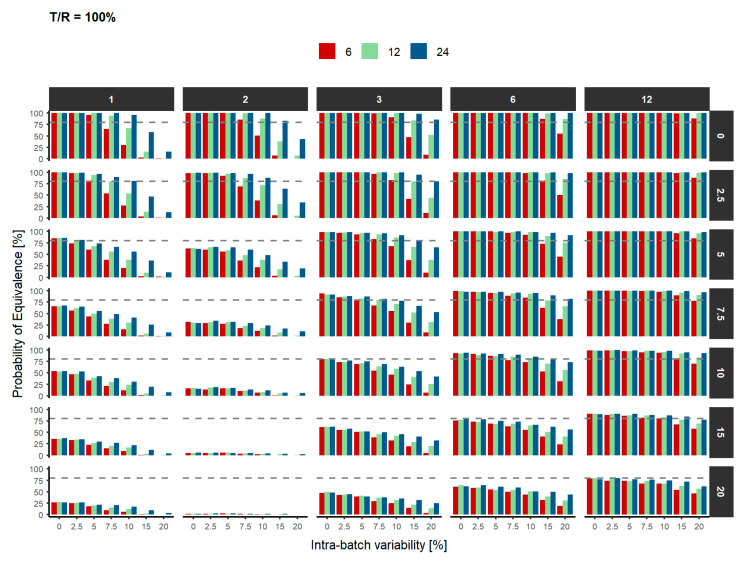
Probability of declaring similarity based on the intra-batch variability and the number of units (red: 6, green: 12 and blue: 24). Top panels represent the number of batches and right panels represent the inter-batch variability. The grey dashed line represents the statistical power to declare equivalence between both products (80%).

Figure 3 summarizes the probability of declaring similarity when the T/R ratio is equal to 100% based on the ABV. The results are stratified by the number of batches and the IBV considered.

**Figure 4:**
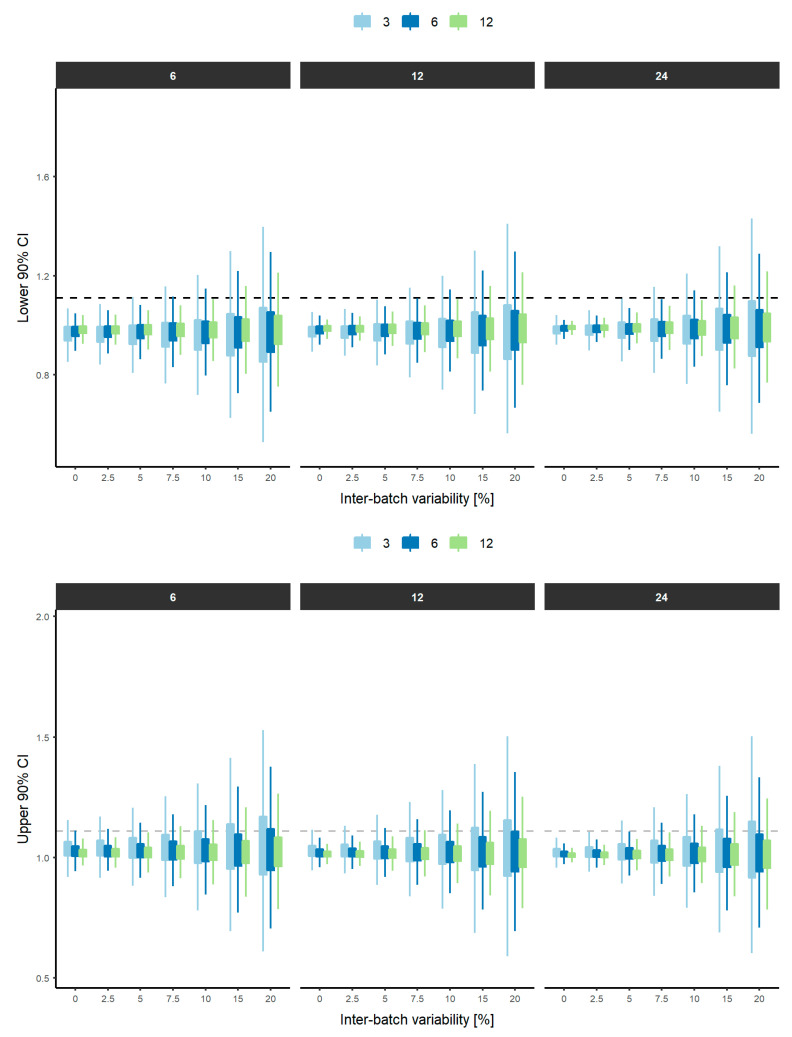
Distribution of the lower (**top**) and upper (**bottom**) 90% confidence intervals from each experiment (*n* = 1000), stratified by the number of units (top) when the T/R ratio is 100%. Light blue, blue and green boxplots represent the lower and upper 90% CI when 3, 6 and 12 batches were considered, respectively. The box represents the interquartile range (IQR: Q1–Q3) and lines represent 1.5*IQR. Dashed grey lines represent the lower (0.9) and upper (1.11) limit of the regulatory acceptance range.

**Figure 5:**
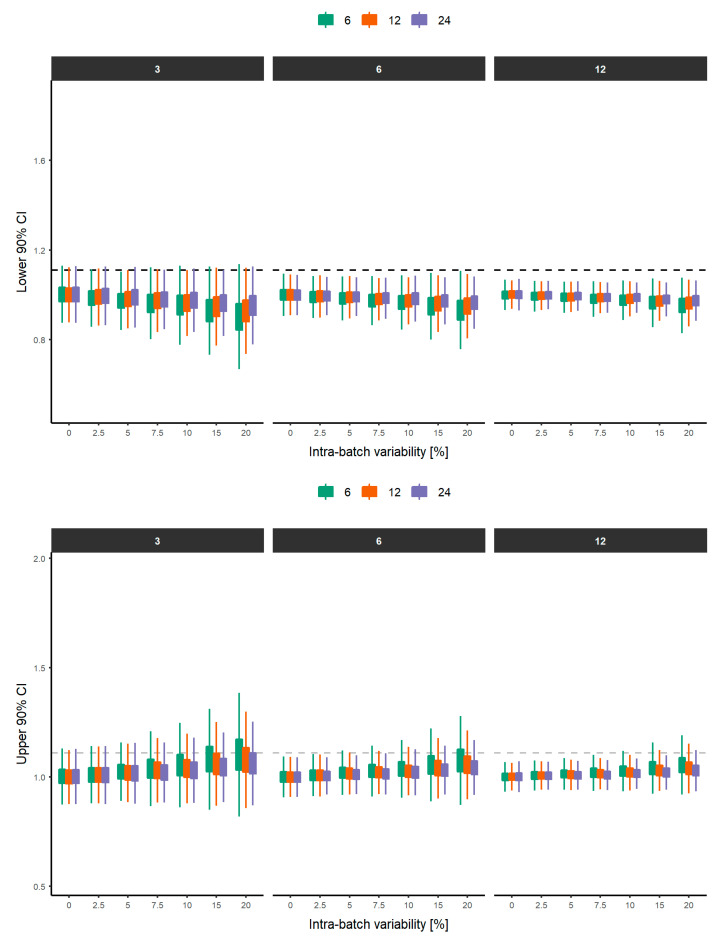
Distribution of the lower (**top**) and upper (**bottom**) 90% confidence intervals from each experiment (*n* = 1000), stratified by the number of batches (**top**). Green, orange and purple boxplots represent the lower and upper 90% CI when 6, 12 and 24 units were considered, respectively. The box represents the interquartile range (IQR: Q1–Q3) and lines represent 1.5*IQR. Dashed grey line represent the lower (0.9) and upper (1.11) limit of the regulatory acceptance range.

The distribution of the lower and upper limits of the 90% CI derived from the bootstrap analysis (*n* = 10,000) for each scenario, assuming a T/R = 100% in the rheological parameter between test and reference products and stratified by N_B_ and N_U_ are depicted in Figure 4 and Figure 5, respectively.

Distributions of the lower and upper limits of the 90% CI narrow as the number of batches increases for the same conditions of IBV. Furthermore, from 2.5% IBV, the differences in the distributions are evident from selecting 3, 6 and 12 batches. This is due to the fact that it is more complex to control the variability in a very small sample (1–3 batches), while if the sample is increased (6–12 batches), a more precise characterization of the variability can be obtained. This trend is independent of the N_U_, demonstrating that IBV is only dependent on the N_B_ considered. This behavior is also visible when ABV and units (6, 12 and 24) are assessed (Figure 5). As long as the ABV increases, a narrower distribution of the 5th and 95th percentiles were obtained when higher N_U_ was considered. As expected, when low ABV exists, a similar distribution of the 5th and 95th percentiles were achieved with 6, 12 and 24 units per batch. However, differences in the distribution start to appear when ABV is equal or greater than 10%, suggesting that 6 units per batch is insufficient to obtain a precise and reliable estimation of the equivalence between two products. This clearly indicates that the influence of ABV can be partially addressed by increasing the number of units per batch, since less variable 5th and 95th percentiles are expected, and therefore, the conclusion of equivalence is less influenced by the sample size. The distributions of the 5th and 95th percentiles with 1 or 2 batches are not shown for simplicity. A similar behavior is observed when differences in the rheological parameter between the test and reference products increase to ±2.5 and ±5% (Supplementary Figures S1 and S2).

## 4. Discussion

This investigation provides the number of batches and the number of replicates per batch required to have enough (>80%) statistical power to show equivalence within a 10% acceptance range where multiple batches are included in the comparison (e.g., for in vitro parameters), in contrast to those comparisons where only batch is tested (e.g., in vivo bioequivalence study). In the case of in vivo studies, it is assumed that the in vivo response (e.g., pharmacokinetic, pharmacodynamic or clinical) would be the same in all manufactured batches, therefore only one batch needs to be tested, but for in vitro parameters (i.e., critical quality attributes) several batches need to be tested because the in vitro outcome varies from batch to batch and between units of a given batch. Importantly, this investigation is not limited to in vitro properties of semisolid dosage forms, but can be applied to any comparison of in vitro parameters that can be assumed to follow a normal/log-normal distribution and where several batches of each product are tested and more than 1 determination is conducted per batch.

In addition, this study also addresses the adequacy of the frequent requirement of testing 3 batches and 12 replicates per batch. As can be seen in Table 1, when the test and reference product are identical (T/R = 1), with 3 batches and 12 replicates per batch there is enough power to show equivalence when IBV is ≤7.5% and ABV is ≤7.5% (≥80% power). In case of higher ABV (e.g., 10%), the IBV should be only 5% to achieve 87% power, and if the ABV is 15%, the IBV should be 2.5% to achieve 80% power. Otherwise, this minimum requirement of 3 batches and 12 replicates per batch would be insufficient, since it only applies to conditions of low IBV and ABV. It is important to highlight that intra- and inter-batch variability higher than 10% has been described in 4 out of 10 rheological parameters that were compared in a previous work, in which ten batches of the reference bethametasone/calcipotriol cream were compared [15,16], and in 2 of the 8 rheological parameters of two oil-in-water emulgels with the same qualitative and quantitative composition containing 2% diclofenac diethylamine, for which 3 and 4 batches were analyzed [19]. In one of these parameters, the observed variability ranged between 25% and 30%, and the droplet diameter exhibited even higher variability.

Furthermore, the comparison of the combination of batches and replicates that produce the same total number of observations (i.e., 3 batches and 24 replicates vs. 6 batches and 12 replicates vs. 12 batches and 6 replicates), shows that 12 batches and 6 replicates are successful in a larger number of scenarios independently of the difference between products. A general recommendation for moderate-to-high variability (≥10%) scenarios (IBV and/or ABV) could be to use 6 batches and 12 units per batch or, in case there is not sufficient number of batches available, 3 batches with 24 units per batch.

The evaluation of the probability of showing equivalence where it is not possible to use three batches because fewer batches are on the market shows that the use of two batches is surprisingly counter-productive. The use of only one batch is impractical, unless the inter-batch variability is known to be very low through the investigation of multiple batches during the pharmaceutical development, since testing only one batch does not allow the characterization of the inter-batch variability. The obtained estimations of the mean would be only accurate if the inter-batch variability were extremely low. When the variability is so low any selected batch can be considered to be representative of future batches to be manufactured for the test product and for any other batch of the reference product in the market. This same assumption is employed when conducting in vivo bioequivalence studies, where it is expected that all batches of the same manufacturer have consistently the same bioavailability. Testing only one batch of test and reference in products with known low inter-batch variability (<2.5%) could be feasible if a large number of replicates per batch were tested. In case of 24 replicates, enough power is achieved for scenarios with 2.5% inter-batch variability only and intra-batch variability of 10% if the ratio between products is 100%, 7.5% intra-batch variability if the ratio is 97.5%, and 5% if the ratio is 95%.

This study also confirms the fact that increases in IBV must be addressed by increasing the number of batches. This trend is irrespective of the T/R ratio simulated (100%, 97.5% and 95%) and the number of units (6, 12 and 24) considered, but only visible when an appropriate characterization of the IBV occurs (i.e., ≥3 batches). For example, in case of identical products or differences of 2.5% or 5%, and ABV of up to 10%, the increase of IBV from 5 to 10% requires testing 6 instead of 3 batches to achieve 80% power to show similarity. This conclusion is supported in Figure 4, since a wider distribution of the 5th and 95th percentiles occurs when 3 batches were considered vs. 6–12 batches, leading to a smaller number of simulations concluding equivalence when both products are actually equivalent (T/R = 100%). This reinforces the fact that IBV cannot be adequately captured when three batches are considered and at least six batches should be used to properly reduce the influence of IBV in the assessment of equivalence between two products. Consequently, the recommendation of the draft guideline [1] to use a minimum of three batches must be interpreted as a minimum to be applied when the inter-batch variability is really low and it must be understood that even in that case it is not estimating the actual inter-batch variability accurately.

Table 1, Table 2 and Table 3 show that in the case of high ABV and IBV, equal to or higher than 20%, there is less than 80% power to show equivalence within an acceptance range of 10%, even if the 12 batches and 24 replicates per batch are tested. The use of more than 24 replicates per batch is possible, but the use of more than 12 batches may be unfeasible, since such a large number of batches may not be available in the European market. Therefore, a widening of the acceptance range is proposed for those cases where there is less than 80% power to show equivalence with a reasonable sample size (e.g., 10–12 batches and 30–50 replicates per batch) even when products are identical. This widening could be applied in the same way as it is applied in pharmacokinetic bioequivalence studies for Cmax in the case of highly variable drugs with intra-subject CV for the reference product higher than 30% [19]. However, in this case the widening should be based on the total variability of the reference, which is the variability to which a patient treated chronically with the reference product is exposed. As in this case the acceptance range is 10%, instead of 20%, the proportionality constant should be different. This proportionality constant would depend on the value of CV where widening starts to be applied, since S_R_, where the limits start to be widened, and S_0_, which defines the scaling proportionality constant, are the same in the Guideline on the investigation of bioequivalence [20]. If it were applied in those cases where the total variability is 10%, the acceptance limits in log-scale would be widened with a proportionality constant of 1.056. If it were applied when the total variability is 20%, the proportionality constant would be 0.532 and if the total CV were 30%, the proportionality constant would be 0.3589, which is similar to the 0.36 value defined by Wellek as a strict one [21]. Further simulation work is necessary to define the proportionality constant for widening of the acceptance range, but for the time being, a proportionality of 0.3589 seems conservative enough.

The magnitude of the IBV and ABV as well as the difference between test and reference differ in the multiple in vitro parameters under comparison. For this reason, the calculation of sample size could be based either individually for each parameter according to the expected variability and the expected difference between products for that parameter, or globally for all parameters based on the maximum sample size required for the parameter that presents greater variability and/or difference. Those decisions can be conditioned by the cost of the analysis and the availability of batches and units. Additionally, as the sponsor has to compare multiple in vitro parameters, multiplicity may cause that some comparison may be non-equivalent simply by chance. The higher number of in vitro parameters, the higher probability to conclude that one of them is not able to show equivalence. Therefore, it would be wise to use the maximum global sample size if affordable. In addition, the multiplicity can be compensated with the possibility to repeat the studies with a higher sample size to achieve conclusive results. In such case, the whole evidence from inconclusive and conclusive studies should be combined to demonstrate consistency.

## 5. Conclusions

In conclusion, this simulation-based analysis shows the influence of inter- and intra-batch variability on the sample size required to conclude on the equivalence between the in vitro characteristics assuming test/reference ratios of 100%, 97.5% and 95%. This work confirms that the influence of inter-batch variability should be addressed by increasing the number of batches and the intra-batch variability could be solved by increasing the number of units/replicates per batch. Testing 3 batches with 12 units per batch seems to be insufficient unless the inter-batch variability is known to be low. Testing 6 batches with 12 units per batch or 3 batches with 24 units per batch would be sufficient to declare the equivalence in most variability scenarios with up to 5% difference between test and reference products.

## Figures and Tables

**Table 1:** Probability of similarity when the population difference between reference and test products is 0 (T/R = 100%). Number of batches and inter-batch variability are depicted in the left margin and number of replicates and intra-batch variability at the top of the table. In red are outlined the scenarios with a probability lower than 80%.

				T/R = 100%																				
				Number of Units																				
				6							12							24						
				Intra-Batch Variability (%)																				
				0	2.5	5	7.5	10	15	20	0	2.5	5	7.5	10	15	20	0	2.5	5	7.5	10	15	20
**Number of batches**	**1**	**Inter-batch variability (%)**	0	100	100	96	65	30	3	1	100	100	100	94	67	16	1	100	100	100	100	96	58	16
			2.5	100	98	82	54	27	3	1	100	98	94	80	54	14	1	100	99	96	90	81	47	13
			5	85	74	60	38	20	2	1	86	81	68	56	38	10	1	86	82	74	66	56	36	11
			7.5	66	57	44	28	16	2	1	66	62	50	39	30	6	1	68	65	56	49	41	26	9
			10	54	47	33	21	12	2	0	53	48	39	30	24	5	1	54	53	43	38	31	20	8
			15	36	33	23	15	9	1	0	36	34	26	20	17	3	1	37	35	30	27	22	12	4
			20	27	25	18	10	6	1	0	28	26	20	16	12	3	0	27	27	22	21	17	10	4
	**2**		0	100	100	100	86	51	7	0	100	100	100	99	88	38	7	100	100	100	100	100	83	43
			2.5	98	98	92	69	38	6	0	98	99	96	87	72	31	5	99	99	98	96	88	64	34
			5	63	60	56	36	22	3	0	64	65	59	48	38	18	3	62	66	65	60	48	34	19
			7.5	32	29	28	18	12	2	0	30	31	31	23	18	9	2	29	34	32	29	24	17	11
			10	16	14	16	10	7	1	0	17	18	16	11	8	5	1	15	19	17	14	12	7	6
			15	5	5	6	3	2	0	0	5	5	6	4	3	2	1	6	6	5	5	4	3	2
			20	2	2	3	2	1	0	0	2	2	2	1	1	1	0	2	3	3	2	2	2	0
	**3**		0	100	100	100	99	91	47	9	100	100	100	100	100	84	52	100	100	100	100	100	98	86
			2.5	100	100	100	96	83	42	11	100	100	100	100	98	80	44	100	100	100	100	100	95	80
			5	99	97	94	83	68	37	10	99	98	96	94	87	66	38	99	99	97	96	92	82	65
			7.5	94	86	81	68	56	30	9	92	87	83	80	71	52	32	92	88	87	82	78	67	53
			10	80	73	69	55	46	25	7	80	73	71	64	59	41	26	82	77	75	69	63	54	42
			15	61	55	51	39	32	19	5	62	55	52	46	42	29	20	62	58	52	50	46	41	32
			20	47	43	40	29	25	15	4	50	44	41	37	32	22	14	48	45	40	38	35	32	25
	**6**		0	100	100	100	100	100	87	55	100	100	100	100	100	99	87	100	100	100	100	100	100	100
			2.5	100	100	100	100	98	82	50	100	100	100	100	100	98	85	100	100	100	100	100	100	98
			5	100	100	100	97	93	73	45	100	100	99	99	99	90	76	100	100	100	100	99	97	92
			7.5	99	98	95	89	85	63	38	99	97	95	94	93	79	66	98	98	98	96	95	90	82
			10	93	91	87	78	73	53	32	92	89	87	85	82	70	56	94	92	91	90	85	80	73
			15	76	73	69	63	55	41	24	77	72	68	69	65	51	41	78	78	75	73	66	62	56
			20	61	58	55	50	44	32	19	64	59	54	54	51	40	31	62	64	61	59	51	50	44
	**12**		0	100	100	100	100	100	99	88	100	100	100	100	100	100	100	100	100	100	100	100	100	100
			2.5	100	100	100	100	100	98	88	100	100	100	100	100	100	99	100	100	100	100	100	100	100
			5	100	100	100	100	100	96	85	100	100	100	100	100	100	96	100	100	100	100	100	100	99
			7.5	100	100	100	99	98	90	78	100	100	99	100	99	96	91	100	100	100	100	100	99	97
			10	99	99	97	95	94	82	70	99	99	97	98	96	92	84	98	100	99	98	98	95	93
			15	90	88	86	82	80	67	58	90	90	87	87	82	77	69	89	90	90	88	87	84	77
			20	79	74	74	68	68	54	46	80	78	73	74	68	63	57	77	80	77	76	75	72	62

**Table 2:** Probability of similarity when the population difference between reference and test products is 2.5%. Number of batches and inter-batch variability are depicted in the left margin and number of replicates and intra-batch variability on the top of the table. In red are outlined the scenarios with a probability lower than 80%.

				T/R = 97.5%																				
				Number of Units																				
				6							12							24						
				Intra-Batch Variability (%)																				
				0	2.5	5	7.5	10	15	20	0	2.5	5	7.5	10	15	20	0	2.5	5	7.5	10	15	20
**Number of batches**	**1**	**Inter-batch variability (%)**	0	100	100	89	59	26	3	1	100	100	99	83	60	17	0	100	100	100	98	88	52	16
			2.5	98	92	78	48	22	3	1	99	95	87	74	52	14	0	99	97	94	86	75	45	13
			5	83	72	58	37	19	2	1	83	77	63	52	39	10	0	82	80	71	64	53	34	10
			7.5	66	57	43	27	15	2	1	65	60	49	38	28	7	1	66	64	54	49	39	26	8
			10	52	45	32	22	12	1	0	52	49	37	30	23	6	1	54	50	42	38	32	21	6
			15	36	32	22	15	9	1	0	36	34	26	20	17	4	1	36	36	29	27	22	14	6
			20	27	24	16	10	7	0	0	28	26	18	15	14	3	1	27	28	23	21	17	10	3
	**2**		0	100	100	96	72	46	7	0	100	100	100	95	77	32	6	100	100	100	100	97	72	38
			2.5	97	93	84	59	37	6	0	96	97	92	81	64	26	4	96	97	95	90	81	58	30
			5	60	56	50	33	20	4	0	57	60	56	44	34	16	3	59	62	60	56	47	32	19
			7.5	31	27	27	16	10	2	0	30	32	29	23	16	9	2	28	33	30	27	22	15	9
			10	15	15	15	9	7	1	0	16	16	16	13	8	5	2	15	18	15	13	12	8	6
			15	5	5	6	3	2	0	0	6	5	7	4	3	2	0	5	7	6	4	4	3	2
			20	2	2	3	2	2	0	0	2	2	3	1	1	1	0	3	3	2	1	1	2	1
	**3**		0	100	100	100	96	82	43	9	100	100	100	100	97	75	44	100	100	100	100	100	94	76
			2.5	100	100	99	91	74	37	10	100	100	100	98	92	70	41	100	100	100	99	97	89	69
			5	98	94	91	78	64	33	8	98	95	92	88	81	58	35	98	96	94	92	87	77	61
			7.5	88	83	79	65	54	29	8	89	84	80	74	68	48	30	90	86	84	79	73	63	49
			10	78	73	67	54	44	25	7	80	73	69	62	57	38	26	80	75	71	66	62	52	42
			15	60	54	51	38	33	20	6	61	55	52	44	41	27	19	61	57	52	48	45	39	31
			20	48	42	40	29	24	15	4	49	42	41	35	32	22	14	48	44	38	38	34	31	25
	**6**		0	100	100	100	100	98	77	49	100	100	100	100	100	94	78	100	100	100	100	100	100	96
			2.5	100	100	100	99	94	73	44	100	100	100	100	100	92	77	100	100	100	100	100	98	92
			5	100	99	98	93	87	67	41	100	99	98	97	96	85	70	99	99	99	99	98	94	86
			7.5	97	96	92	87	78	59	36	97	94	92	90	89	76	62	97	97	94	94	91	85	78
			10	90	88	83	78	71	53	32	91	87	82	83	81	65	54	91	90	88	87	83	76	68
			15	74	72	65	61	53	40	24	75	72	67	66	63	52	41	76	76	73	72	63	60	54
			20	62	58	52	49	42	31	18	62	58	53	54	50	40	33	63	64	60	58	50	47	42
	**12**		0	100	100	100	100	100	95	80	100	100	100	100	100	100	97	100	100	100	100	100	100	100
			2.5	100	100	100	100	100	93	77	100	100	100	100	100	100	96	100	100	100	100	100	100	100
			5	100	100	100	99	98	88	74	100	100	100	100	100	98	91	100	100	100	100	100	99	97
			7.5	100	100	99	97	94	83	70	100	99	98	98	96	92	86	99	100	99	100	99	97	93
			10	97	96	94	92	88	77	64	97	97	96	94	92	87	79	97	96	97	97	95	92	87
			15	88	85	83	79	76	64	54	89	86	84	84	80	76	66	88	87	86	85	83	82	73
			20	78	74	71	68	65	52	45	77	75	71	72	66	63	56	76	76	74	73	72	71	61

**Table 3:** Probability of similarity when the population difference between reference and test products is 5%. Number of batches and inter-batch variability are depicted in the left margin and number of replicates and intra-batch variability on the top of the table. In red are outlined the scenarios with a probability lower than 80%.

				T/R = 95%																				
				Number of Units																				
				6							12							24						
				Intra-Batch Variability (%)																				
				0	2.5	5	7.5	10	15	20	0	2.5	5	7.5	10	15	20	0	2.5	5	7.5	10	15	20
**Number of batches**	**1**	**Inter-batch variability (%)**	0	100	99	68	42	19	2	0	100	100	89	59	42	12	1	100	100	99	82	63	37	13
			2.5	93	79	62	38	21	3	1	94	87	70	55	42	10	1	94	90	80	71	57	35	11
			5	76	65	49	32	16	2	0	76	69	57	46	34	10	1	76	72	64	58	47	29	9
			7.5	61	52	39	25	14	1	0	63	57	44	37	27	8	0	62	58	48	45	37	25	7
			10	49	43	31	20	12	2	0	52	47	36	29	22	6	0	50	49	40	37	30	20	7
			15	35	30	22	15	8	1	0	37	33	25	21	16	5	0	35	36	28	25	22	15	5
			20	26	24	16	11	7	1	0	28	26	19	16	13	3	0	27	27	22	21	17	11	4
	**2**		0	100	100	77	48	30	6	0	100	100	96	72	51	22	4	100	100	100	94	78	45	26
			2.5	84	78	61	42	24	3	0	83	83	70	56	44	17	3	82	84	80	71	59	39	20
			5	50	46	42	27	16	2	0	46	50	45	37	28	13	2	46	51	46	42	37	24	13
			7.5	26	25	24	15	10	1	0	26	27	27	21	16	8	1	27	28	26	23	18	14	7
			10	14	12	13	8	6	1	0	15	14	15	11	9	5	1	13	16	13	13	10	7	5
			15	5	5	5	3	2	0	0	6	5	7	4	4	2	0	5	7	5	4	3	3	2
			20	2	3	3	1	2	0	0	3	2	3	2	2	1	0	3	3	2	2	1	1	1
	**3**		0	100	100	97	78	56	28	9	100	100	100	94	79	48	30	100	100	100	100	95	73	52
			2.5	100	98	89	71	52	28	8	100	98	94	85	73	49	31	100	99	97	94	85	68	50
			5	90	85	78	63	50	28	7	92	85	82	72	65	47	29	92	88	84	80	73	60	48
			7.5	80	76	70	57	47	26	7	82	77	72	64	57	42	26	81	78	72	71	64	54	41
			10	72	67	62	50	41	24	6	74	69	64	56	50	36	24	72	68	64	61	55	47	37
			15	58	52	50	38	31	19	4	58	52	49	43	38	26	18	57	53	49	46	42	36	30
			20	46	41	41	29	22	14	4	46	43	39	33	30	20	14	46	44	38	37	34	30	23
	**6**		0	100	100	100	95	83	52	34	100	100	100	100	96	74	54	100	100	100	100	100	94	77
			2.5	100	100	98	88	75	55	33	100	100	99	96	92	72	54	100	100	100	99	98	88	70
			5	97	95	90	81	70	51	32	98	95	91	88	82	68	52	97	97	94	92	88	79	64
			7.5	89	86	81	74	63	48	32	91	87	80	80	75	62	50	90	90	86	84	78	71	60
			10	81	79	73	67	58	44	30	84	80	74	72	69	56	46	83	83	80	76	71	65	55
			15	70	66	59	54	49	37	24	69	66	62	60	57	45	37	72	69	68	65	58	53	47
			20	59	56	49	46	39	32	18	59	56	52	51	48	37	29	61	59	56	56	47	45	39
	**12**		0	100	100	100	100	96	77	58	100	100	100	100	100	94	78	100	100	100	100	100	100	96
			2.5	100	100	100	99	94	72	53	100	100	100	100	99	90	76	100	100	100	100	100	99	92
			5	100	99	97	93	86	70	52	100	99	98	98	94	85	72	99	100	99	99	98	94	85
			7.5	96	94	91	86	79	66	52	96	95	92	90	86	78	68	96	95	94	94	91	88	78
			10	91	87	84	81	74	62	50	92	88	86	84	80	73	63	91	88	88	87	84	82	72
			15	81	78	75	71	65	54	45	81	77	75	74	69	64	56	80	78	76	76	74	71	63
			20	71	68	65	62	56	47	40	71	67	66	66	61	56	50	70	70	68	68	66	62	54

## Supplementary Materials

The following are available online at https://www.mdpi.com/1999-4923/12/12/1159/s1, Figure S1: Distribution of the lower (top) and upper (bottom) 90% confidence intervals from each experiment (*n* = 1000), stratified by the number of units (top) when the T/R ratio is 97.5%. Figure S2: Distribution of the lower (top) and upper (bottom) 90% confidence intervals from each experiment (*n* = 1000), stratified by the number of batches (top) when the T/R ratio is 97.5%. Figure S3: Distribution of the lower (top) and upper (bottom) 90% confidence intervals from each experiment (*n* = 1000), stratified by the number of units (top) when the T/R ratio is 95%. Figure S4: Distribution of the lower (top) and upper (bottom) 90% confidence intervals from each experiment (*n* = 1000), stratified by the number of batches (top) when the T/R ratio is 95%.
