# Supplementary material for: Influence of Inter- and Intra-Batch Variability on the Sample Size Required for Demonstration of Equivalent Microstructure of Semisolid Dosage Forms
Source: Pharmaceutics. 2020 Nov 28;12(12):1159. doi: 10.3390/pharmaceutics12121159 (PMC7760601; doi:10.3390/pharmaceutics12121159)
Supplement: Supplementary file 1 [file pharmaceutics-12-01159-s001.pdf]

pharmaceutics

Supplementary Material: Influence of Inter- and Intra-Batch Variability on the Sample Size Required for Demonstration of Equivalent Microstructure of Semisolid Dosage Forms

Zhengguo Xu, Víctor Mangas-Sanjuán, Matilde Merino-Sanjuán, Virginia Merino and Alfredo García-Arieta

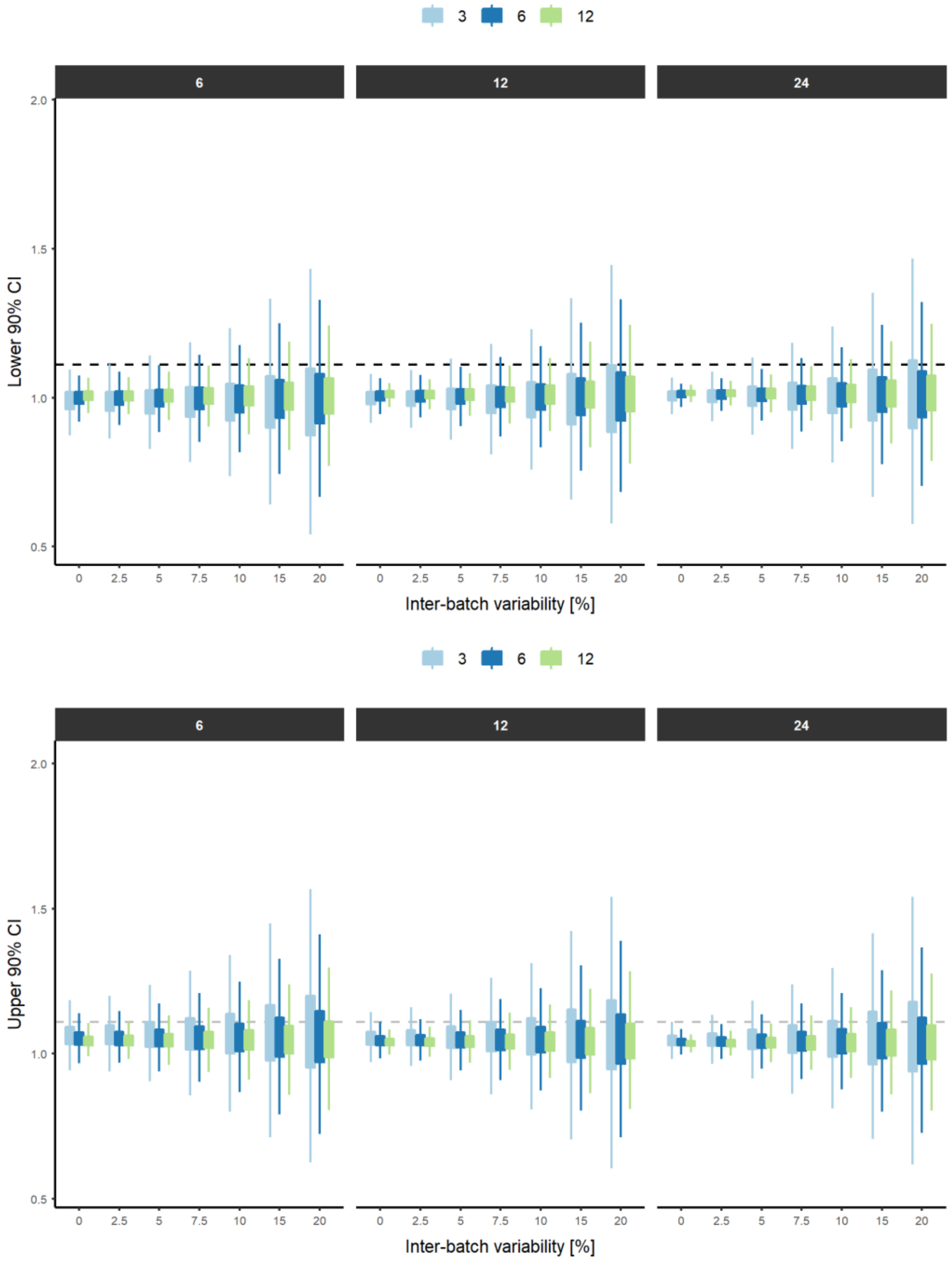

Figure S1. Distribution of the lower (top) and upper (bottom) 90% confidence intervals from each experiment (n=1000), stratified by the number of units (top) when the T/R ratio is 97.5%. Light blue,

blue and green boxplots represent the lower and upper 90% CI when 3, 6, and 12 batches were considered, respectively. The box represents the interquartile range (IQR: Q1-Q3) and lines represent 1.5*IQR. Dashed grey line represent the lower (0.9) and upper (1.11) limit of the regulatory acceptance range.

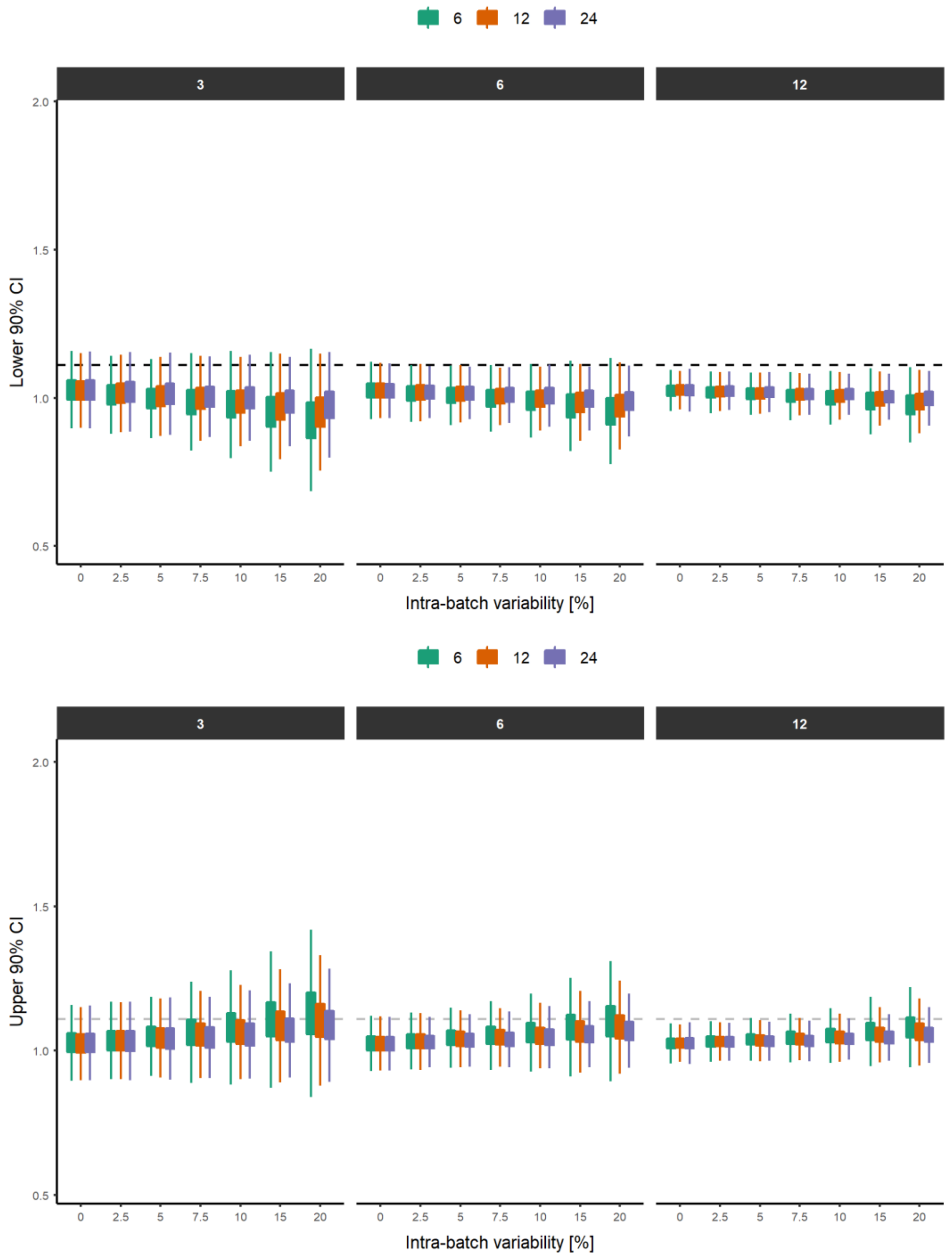

Figure S2. Distribution of the lower (top) and upper (bottom) 90% confidence intervals from each experiment (n=1000), stratified by the number of batches (top) when the T/R ratio is 97.5%. Green, orange and purple boxplots represent the lower and upper 90% CI when 6, 12, and 24 units were considered, respectively. The box represents the interquartile range (IQR: Q1-Q3) and lines represent 1.5*IQR. Dashed grey line represent the lower (0.9) and upper (1.11) limit of the regulatory acceptance range.

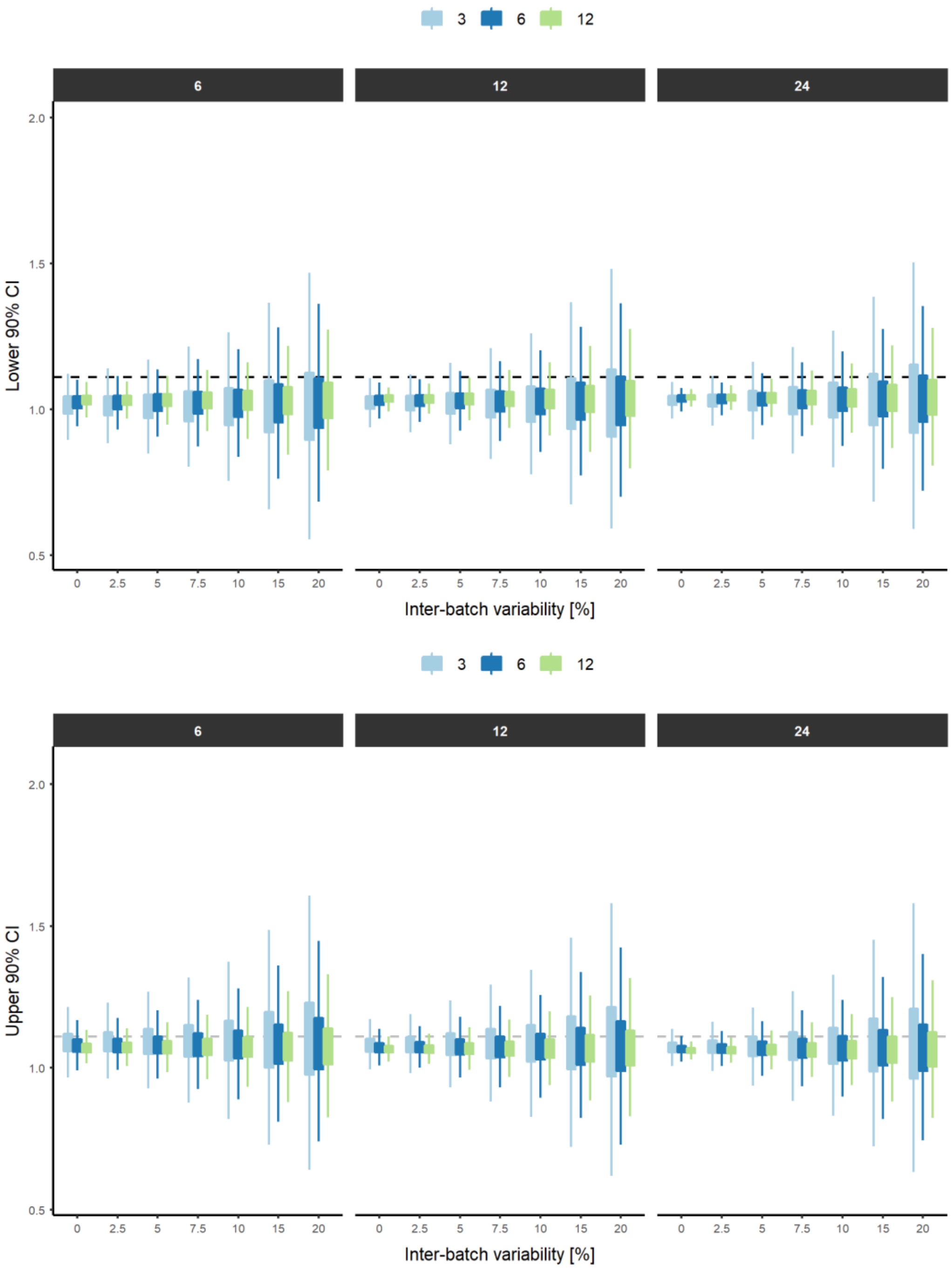

Figure S3. Distribution of the lower (top) and upper (bottom) 90% confidence intervals from each experiment (n=1000), stratified by the number of units (top) when the T/R ratio is 95%. Light blue, blue and green boxplots represent the lower and upper 90% CI when 3, 6, and 12 batches were considered, respectively. The box represents the interquartile range (IQR: Q1-Q3) and lines represent 1.5*IQR. Dashed grey line represent the lower (0.9) and upper (1.11) limit of the regulatory acceptance range.

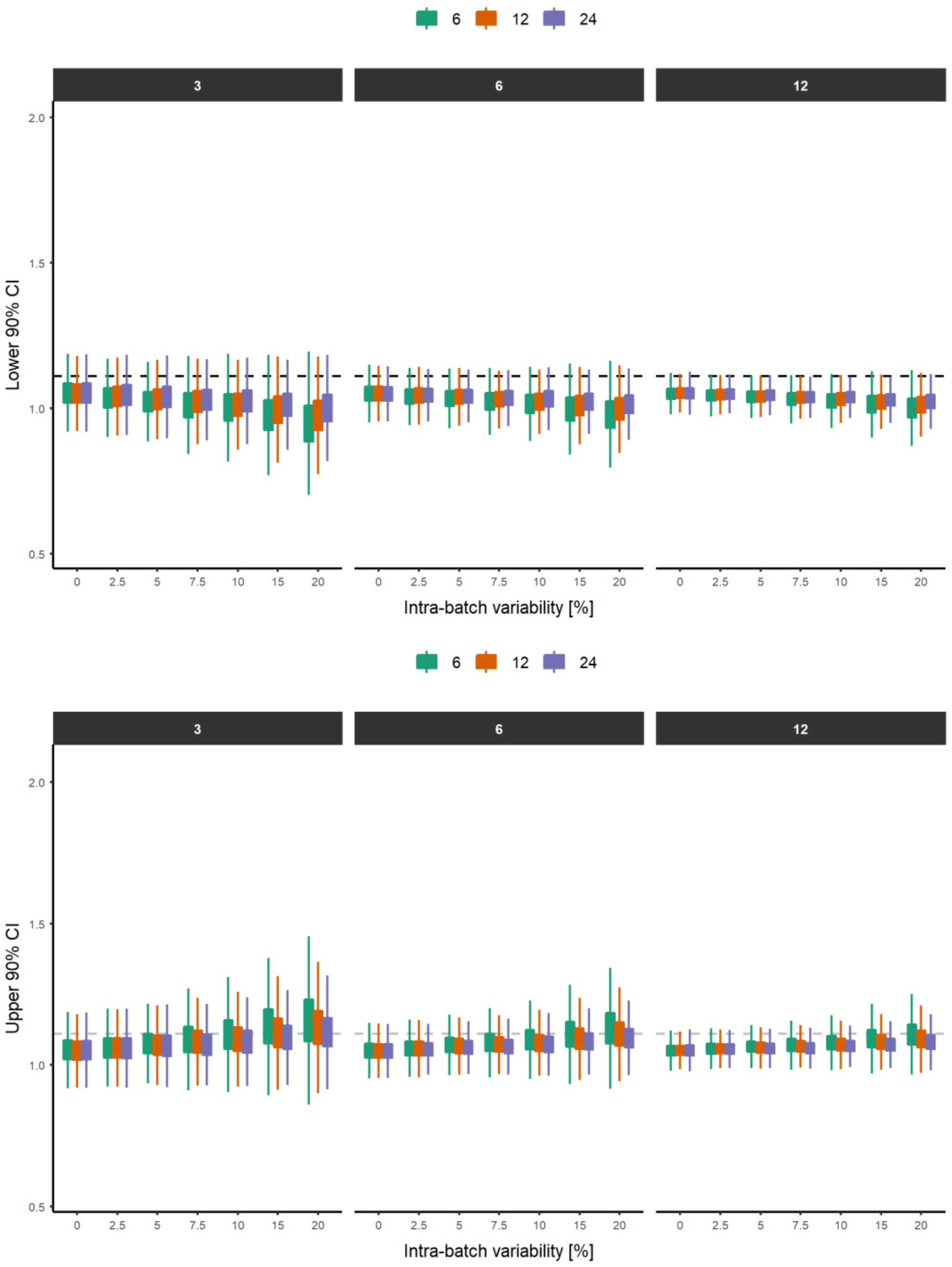

Figure S4. Distribution of the lower (top) and upper (bottom) 90% confidence intervals from each experiment (n=1000), stratified by the number of batches (top) when the T/R ratio is 95%. Green, orange and purple boxplots represent the lower and upper 90% CI when 6, 12, and 24 units were considered, respectively. The box represents the interquartile range (IQR: Q1-Q3) and lines represent 1.5*IQR. Dashed grey line represent the lower (0.9) and upper (1.11) limit of the regulatory acceptance range.
